# Molecular Cloning and Characterization of the Human ErbB4 Gene: Identification of Novel Splice Isoforms in the Developing and Adult Brain

**DOI:** 10.1371/journal.pone.0012924

**Published:** 2010-09-23

**Authors:** Wei Tan, Michael Dean, Amanda J. Law

**Affiliations:** 1 Basic Science Program, Science Application International Corporation - Frederick, National Cancer Institute at Frederick, Frederick, Maryland, United States of America; 2 Laboratory of Experimental Immunology, Cancer and Inflammation Program, Center for Cancer Research, National Cancer Institute at Frederick, Frederick, Maryland, United States of America; 3 Clinical Brain Disorders Branch, Intramural Research Program, National Institute of Mental Health, National Institutes of Health, Bethesda, Maryland, United States of America; Duke University, United States of America

## Abstract

ErbB4 is a growth factor receptor tyrosine kinase essential for neurodevelopment. Genetic variation in ErbB4 is associated with schizophrenia and risk-associated polymorphisms predict overexpression of ErbB4 CYT-1 isoforms in the brain in the disorder. The molecular mechanism of association is unclear because the polymorphisms flank exon 3 of the gene and reside 700 kb distal to the CYT-1 defining exon. We hypothesized that the polymorphisms are indirectly associated with ErbB4 CYT-1 via splicing of exon 3 on the CYT-1 background. We report via cloning and sequencing of adult and fetal human brain cDNA libraries the identification of novel splice isoforms of ErbB4, whereby exon 3 is skipped (del.3). ErbB4 del.3 transcripts exist as CYT-2 isoforms and are predicted to produce truncated proteins. Furthermore, our data refine the structure of the human ErbB4 gene, clarify that juxtamembrane (JM) splice variants of ErbB4, JM-a and JM-b respectively, are characterized by the replacement of a 75 nucleotide (nt) sequence with a 45-nt insertion, and demonstrate that there are four alternative exons in the gene. Our analyses reveal that novel splice variants of ErbB4 exist in the developing and adult human brain and, given the failure to identify ErbB4 del.3 CYT-1 transcripts, suggest that the association of risk polymorphisms in the ErbB4 gene with CYT-1 transcript levels is not mediated via an exon 3 splicing event.

## Introduction

Alternative splicing is a key element in gene regulation that increases proteome diversity and the coding potential of the human genome. Evidence from expressed sequence tag, cDNA, genome-wide tiling and splicing microarray datasets demonstrate that alternative splicing occurs in >90% of genes [1]–[3]. Alternative splicing is especially prevalent in the brain which may account for the high incidence of genetic brain disorders.

Linkage and association analyses have identified neuregulin 1 and its receptor ErbB4 as susceptibility genes for schizophrenia [4]–[7] and recently partial deletions of the ErbB4 gene have been reported in the disorder [8]. ErbB4 is a member of the receptor tyrosine kinase family, which also includes epidermal growth factor receptor (EGFR/HER1/ErbB1), HER2/ErbB2, and HER3/ErbB3. The human ErbB4 gene spans 1.16 Mb (megabases) on chromosome 2q34 and has been annotated to consist of 28 exons. To date, four structurally and functionally distinct splice isoforms of the human ErbB4 gene have been described. The JM-ErbB4 isoforms differ within their extracellular juxtamembrane domain annotated as based on differential inclusion of exon 15 (JM-a) or exon 16 (JM-b), which renders them susceptible or resistant, respectively, to processing by matrix metalloproteases [9], [10]. The ErbB4-CYT isoforms arise from additional splicing of the cytoplasmic tail region based on the inclusion (CYT-1) or exclusion (CYT-2) of exon 26, encoding 16 amino acids [11].

Previous studies have demonstrated that a haplotype consisting of three single nucleotide polymorphisms (SNPs; rs707284, rs839523 and rs7598440) in the human ErbB4 gene is associated with increased risk for schizophrenia, augmented expression of the ErbB4 CYT-1 phosphoinositide 3-kinase (PI3K)-linked isoform in the brain of patients and altered frontotemporal structural connectivity in normal humans measured with magnetic resonance imaging (MRI) techniques [6], [7], [12]. The mechanism by which the risk SNPs influence ErbB4 CYT-1 expression is at present unclear, primarily because the SNPs are intronic and reside 700 kilobases (kb) 5′ to the CYT-1 specific exon. Interestingly, the risk SNPs flank both the 5′ and 3′ extent of exon 3 and thus may have potential to affect splicing of this exon as part of the splicing code [13].

In the present study we tested the hypothesis that ErbB4 risk SNPs are indirectly associated with CYT-1 expression in the brain [7] through tagging of a population of exon 3 spliced isoforms, to which the polymorphisms are proximal. We report the identification of novel ErbB4 exon 3-skipping (del.3) splice variants which occur on the CYT-2 isoform background, in both fetal and adult human brain, demonstrating for the first time that the ErbB4 exon 3 is an alternative exon. Gene expression analysis reveals that ErbB4 del.3 mRNAs in the fetal brain are primarily JM-b/CYT-2 isoforms with lower levels of JM-a/CYT-2, whereas those expressed in the adult are exclusively JM-a/CYT-2 isoforms, demonstrating that these splice isoforms are developmentally regulated.

Finally, we show that JM-a and JM-b splice isoforms of ErbB4 are distinguished by substitution of a 75-nt sequence with a 45-nt insertion, respectively and that contrary to the original studies [9], [10], the JM-b exon maps downstream of exon 15, but upstream of exon 16. We therefore, propose to name this 45-nt sequence, exon E15b. Both exons E15b and 16 are mutually exclusive and contrary to earlier characterization of the gene, exon 15 is constitutive.

Together these data provide novel insight into the molecular regulation of ErbB4 alternative splicing in human brain across development, clarify the exon-intron structure of ErbB4 and suggest that a mechanism underlying association of risk polymorphisms in ErbB4 with schizophrenia and CYT-1 expression [7] does not involve exon 3 skipping.

## Results

### A del.3 transcript variant of the human ErbB4 gene is expressed in human fetal brain

It has previously been reported that three intronic risk SNPs (rs7598440, rs707284, rs839523) which comprise a core-risk haplotype are associated with increased genetic risk for schizophrenia and augmented expression of a family of ErbB4 CYT-1 transcripts in the brain of patients with the disorder [6], [7]. The SNPs are physically mapped to the flanking intronic regions of exon 3 (Figure S1A) and may represent intronic *cis*-acting elements of the splice code [13]. Based on this, we examined whether transcript variants of ErbB4, with exon 3 skipped (del.3), exist in human brain. Using a forward primer in exon 1 and two reverse primers in exons 4 and 5, we identified in human fetal brain, cDNA transcripts that consist of exon 1 and exon 2, spliced to exon 4 (Figure S1B). These experiments demonstrate that exon 3 is a novel alternatively spliced exon of the ErbB4 gene.

### ErbB4 del.3 transcripts exist on the CYT-2 isoform background in human fetal brain

**Figure 1:**
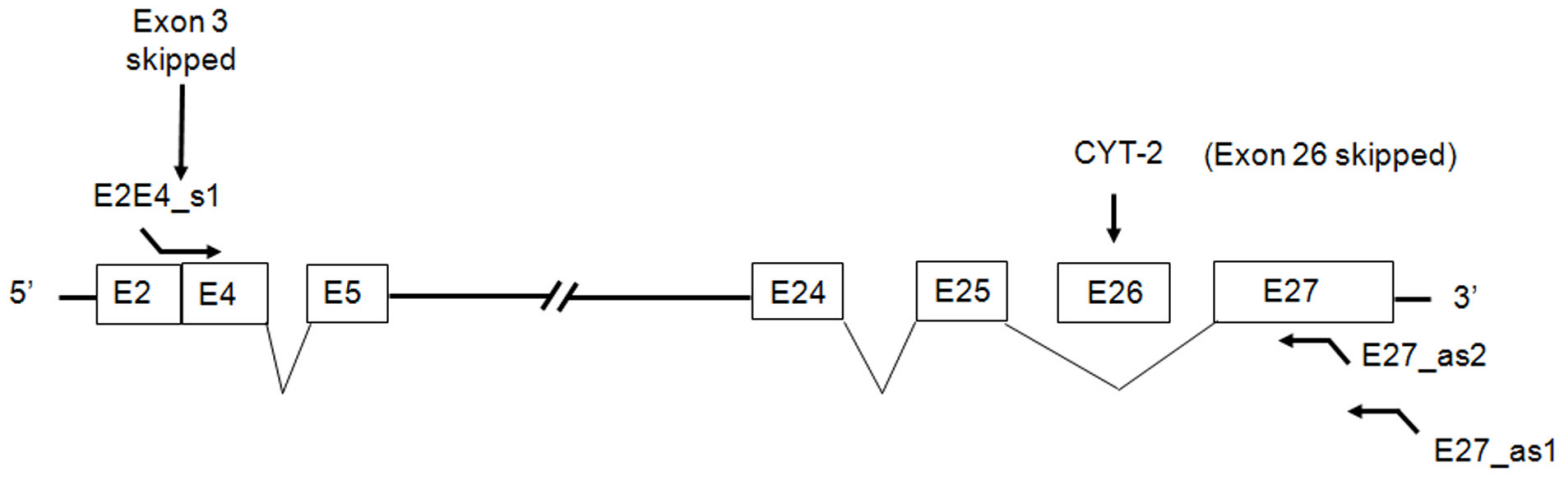
Organization of the ErbB4 del.3/CYT-2 transcript cloned from the human fetal brain. Horizontal lines with “//” sign represent exons from E6 to E23. Exons are shown in the order in which they occur in the transcript. The skipped exons are indicated. The length of each exon is not drawn to scale The PCR primers are indicated as forward or reverse bent arrows, respectively. Names of the primers are shown.

In order to determine whether ErbB4 del.3 transcripts expressed in human brain exist on the CYT-1 or CYT-2 isoform background we designed a forward primer, (E2E4_s1), which spans the junction of exons 2 and 4 combined with reverse primers in exon 27. The difference between the ErbB4 CYT-1 and CYT-2 isoforms lies in exon 26, with its inclusion or exclusion, respectively. In fetal human brain, we amplified a PCR product of approximately 2.73 kb (Figure S2A). After cloning and sequencing 59 cDNA clones, we confirmed that all clones had ErbB4 exon 3 skipped. Deep sequencing of the junction of exons 2 and 4, as well as the region between exons 25 and 27 revealed that the del.3 transcripts all have exon 26 excluded (Figure 1). This result suggests that ErbB4 del.3 transcripts exist in the human fetal brain as del.3/CYT-2 ErbB4 isoforms.

### ErbB4 del.3 exists as a del.3/JM-b/CYT-2 isoform in human fetal brain

The coding region of full-length ErbB4 cDNA was originally identified from sequencing of plasmids containing ErbB4 inserts from a human MDA-MB-453 breast cancer cell line and pEV7-HER4, from fetal human brain tissue [9]. Sequencing revealed the existence of two ErbB4 isoforms that differed by insertion of either 23 or 13 alternative amino acids in the extracellular juxtamembrane region. The 23-amino acid form was termed, JM-a, and the 13-amino acid form, JM-b. These sequences were later mapped to exon 16 and exon 15 of the human ErbB4 gene (10), whereby JM-a was ascribed to exon 16, and its alternative sequence JM-b, to exon 15.

**Figure 2:**
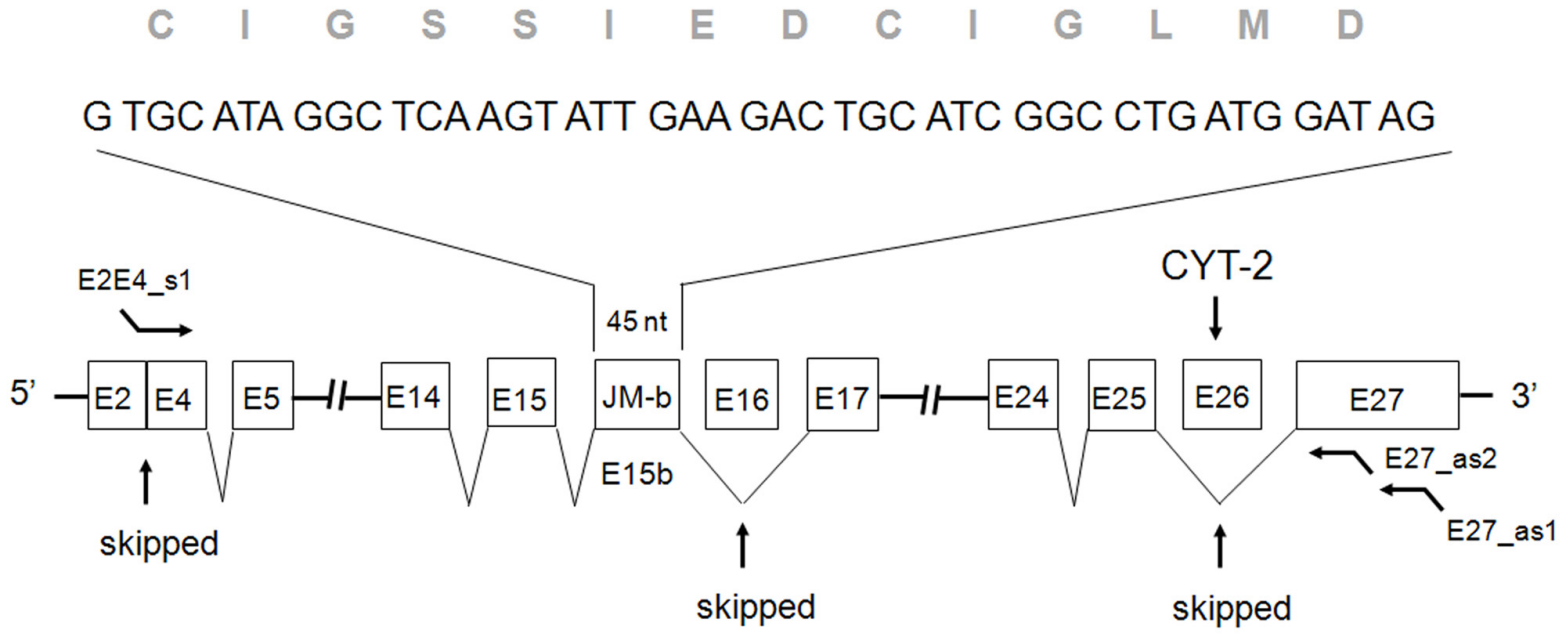
The ErbB4 del.3/CYT2 variant exists as a JM-b isoform in the human fetal brain. Open boxes represent exons. The horizontal lines with the first “//” sign represent exons E6 to E13. Horizontal lines with the second “//” sign represent exons from E18 to E23. The PCR primers are indicated as forward or reverse bent arrows, respectively. Names of the primers are shown. The three spliced exons (exons 3, 16, and 26) are indicated. The 14 aa sequence encoded by the 45-nt, specific for the JM-b isoform, is shown.

**Figure 3:**
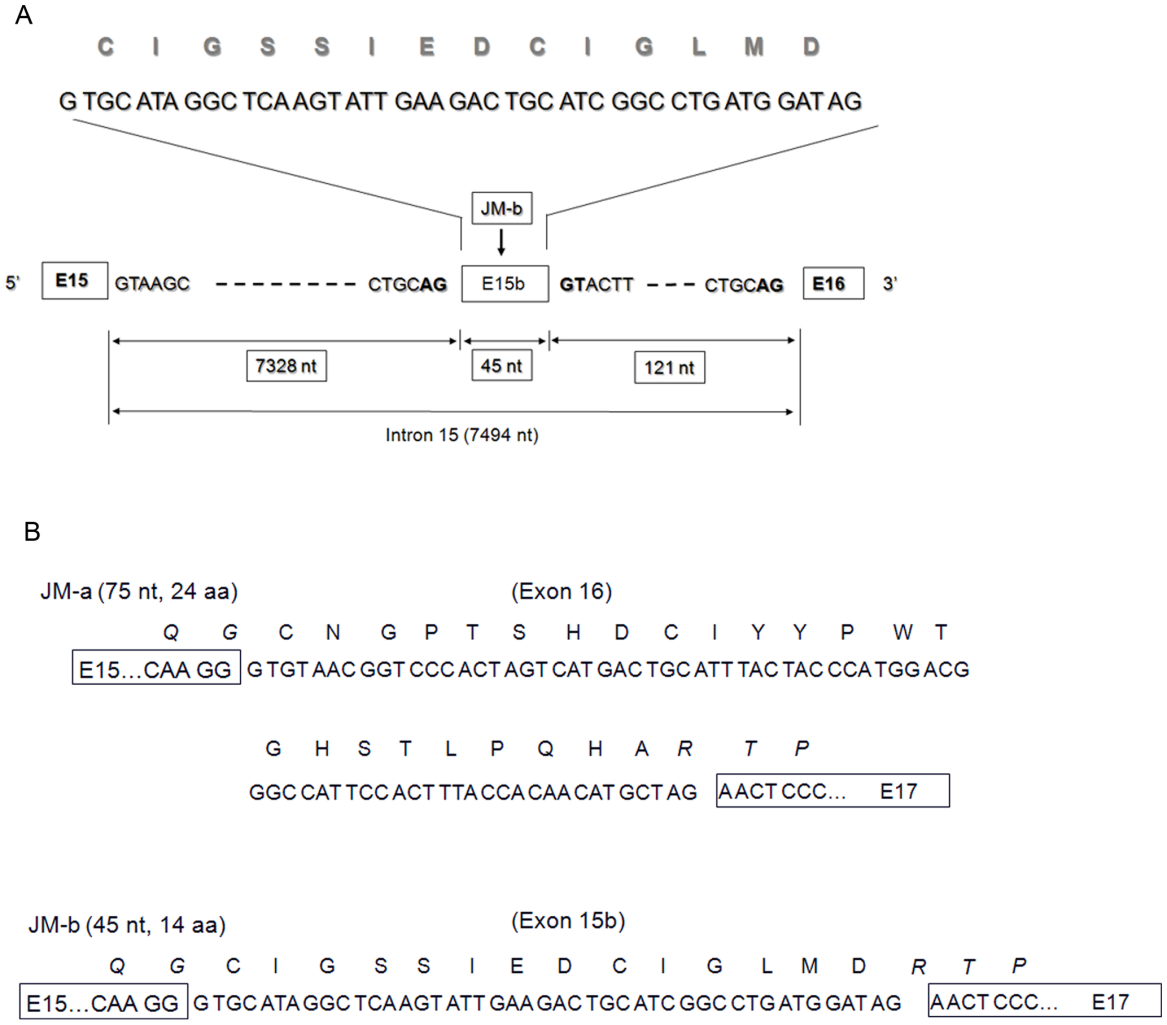
A. Partial exon-intron structure in a region of the ErbB4 gene containing exon 15, intron 15, and exon 16. The 45-nt sequence unique to the JM-b isoform maps to the 3′ region of the intron 15, 121-nt upstream of exon 16 of the ErbB4 gene. Exons 15, 15b and 16 are shown, respectively. Partial intronic sequences, downstream of the exon 15, upstream of the exon 16, as well as upstream and downstream of the exon 15b are shown. Dashed lines represent intron 15 sequence. The 14 aa encoded by the 45-nt sequence, specific for the JM-b isoform, is shown. The length of the intron 15 of the ErbB4 gene is indicated. B. Comparison of JM-a and JM-b sequences showing the nucleotide sequences specific for JM-a or JM-b isoform and amino acids encoded by each exon. The glycine residue maps to the end of exon 15 of human ErbB4 sequence NM_005235.2. The first cysteine is common to both JM-a and JM-b but is encoded by different nucleotide sequences belonging to exons 16 and 15b, respectively.

We have demonstrated that the del.3/CYT-2 isoform, but not the del.3/CYT-1 isoform, exist in human fetal brain. In order to determine whether del.3/CYT-2 transcripts exist as JM-a or JM-b isoforms we deep sequenced the region between exons 5 and 24 in del.3/CYT-2 derived clones, paying particular attention to the sequences between the exons 14 and 17. We observed that exon 16 was skipped in all del.3/CYT-2 transcripts sequenced from fetal brain, demonstrating that del.3/CYT-2 variants in fetal brain exist primarily as JM-b transcripts (Figure 2). Interestingly, in contrast to the original published ErbB4 sequence and exon structure [10], we observed one major difference. Sequencing of the ErbB4 coding region of del.3/JM-b/CYT-2 revealed a 45-nt insert, which is homologous to the sequence encoding 14 amino acids [CIGSSIEDCIGLMD], specific to the JM-b isoform (Figure 3; Figure S3A). However, this insertion represents an alternative exon located 3′ of exon 15 (Figure S3A). The physical location of the JM-b insertion is shown in Figure 2.

### Human ErbB4/JMb isoforms are defined by inclusion of a 45-nt exon (E15b) encoding 14 amino acids

The ErbB4/JM-b isoform was originally defined by an in-frame alteration in which 69-nt in the extracellular juxtamembrane domain of the ErbB4 gene was replaced with an unrelated sequence of 39-nt (9). These sequences were later mapped to exon 16 or exon 15 of the ErbB4 gene, respectively [10]. Detailed visual analysis of our sequences (Figure S3A) and mapping to the human genome using the NCBI and UCSC databases (Assembly; Mar.2006 NCBI36/hg18), reveals that a 45-nt sequence specific for the JM-b isoform does not match to any sequence in exon 15 (155 bp) of the human ErbB4 gene (NM_005235.2). Instead, this 45-nt sequence is 100% identical to a downstream region in intron 15 (Figure 3A). Specifically, this sequence is located close to the 3′ end of ErbB4 intron 15 (chr2: 212,725,219-212725260), and is flanked by consensus splice sites at the 5′ and 3′ ends (Figure 3A). We next compared the mRNA sequence of the JM-a/CYT-1 isoform to that of the JM-b/CYT-1 isoform. Interestingly, in contrast to previous reports (10) these two isoforms have identical sequences in exon 15 (Figure S3A and 3B). The only difference in sequence between the two transcripts is that the JM-a isoform is characterized by the insertion of a 75-nt sequence derived from exon 16, whereas JM-b is encoded by the alternative 45-nt sequence. Our observation that the JM-a specific sequence is 75-nt, rather than 69-nt, as previously reported [9] is consistent with the current reference sequences (NM_001042599.1, NM_005235.2).

Therefore, our sequencing data demonstrate that the human ErbB4 JM-a or JM-b splice isoform is actually invariable in exon 15 and exon 17 (Figure 3B; Figure S3A and 3B). At the genomic DNA level, there are two alternative exons between exons 15 and 17. Given the fact that the 45-nt sequence encoding the JM-b isoform is present in intron 15 upstream of exon 16, and that this 45-nt can be spliced out to generate a JM-b isoform, we propose to term this 15b (E15b) to distinguish it from the upstream constitutive exon 15 (155 bp; Figure 3). These results clarify which internal exon encodes the ErbB4 JM-b isoform, and demonstrate that the ErbB4 JM-b isoform is not encoded by exon 15, which is constitutive, but is instead encoded by a downstream alternative exon E15b, located between the exons 15 and 16 (Figure 3A). Hence, exon 15b and exon 16 are mutually exclusive alternative exons (Figure 3B).

In addition, we demonstrate through sequencing and BLAST-mapping to the human genome using the NCBI and UCSC databases (Assembly; Mar.2006 NCBI36/hg18) that ErbB4 E15b encodes 14 (rather than 13, [9]) unique amino acids specific to the JM-b isoform, whereas exon 16 encodes 24 (rather than 23 [9]) amino acids specific to the JM-a isoform (Figure 3B). This discrepancy appears to arise because the amino acid residue cysteine (C) located at the N-terminus of both JM-a and JM-b was observed to be common to both isoforms in the original study [9]. Indeed, at the amino acid level this is the case; however, the cysteine in the JM-a isoform is encoded by the DNA sequence TGT (UGU in mRNA), whereas the cysteine in JM-b is encoded by TGC (UGC in mRNA; Figure 3B). This sequence is consistent with what was reported in the original study by Elenius and colleagues [9], but limited sequence information regarding exon-intron structure of the human ErbB4 gene at that time, likely contributed to the interpretation that the glycine and cysteine at the N-terminus of JM-a or JM-b isoform were encoded by the same constitutive exon. Our data demonstrate that, the glycine and cysteine are encoded by *separate* exons (exon 15 plus exon 16; or exon 15 plus exon 15b). The glycine and cysteine in the JM-a isoform are located at the boundary of exons 15 and 16. These two amino acid residues are encoded by a GG dinucleotide from exon 15, together with G TGT from exon 16. In contrast, the glycine and cysteine in JM-b isoform are located at the boundary of exons 15 and 15b (Figure 3B).

As a result, we demonstrate that the human ErbB4 gene contains 29, rather than 28 exons. Exons 3, 15b, 16, and 26 are alternative and exons 15b and 16 are mutually exclusive. We have clarified that the juxtramembrane splicing event giving rise to JM-a and JM-b results from an in-frame alteration in which 75-nt encoding 24 amino acids is replaced with an unrelated 45-nt sequence encoding 14 amino acids.

### ErbB4 del.3 is identified as a JM-a/CYT-2 isoform in adult brain

We have shown through PCR screening and cloning that ErbB4 del.3 transcripts in human fetal brain are expressed exclusively on the JM-b/CYT-2 isoform background. To determine whether the del.3/CYT-2 transcript is expressed in the adult human brain we performed nested PCR for amplification of the ErbB4 exon 3-skipping transcript (Figure S2B). Sequencing of ErbB4 cDNA clones derived from adult brain revealed that both exons 3 and 26 are skipped, similar to that found in fetal brain. Again, we did not detect ErbB4 del.3/CYT-1 transcripts in our screen of the adult human brain cDNA library. Interestingly, all clones also lacked exon 15b, but included exon 16, characteristic of the JM-a isoform (Figure S3B and S4). Therefore, in contrast to the human fetal brain, the ErbB4 del.3 transcripts cloned in the adult brain were preferentially expressed on the JM-a/CYT-2 isoform background.

### Expression of ErbB4 del.3 mRNA transcripts in adult and fetal human brain

**Figure 4:**
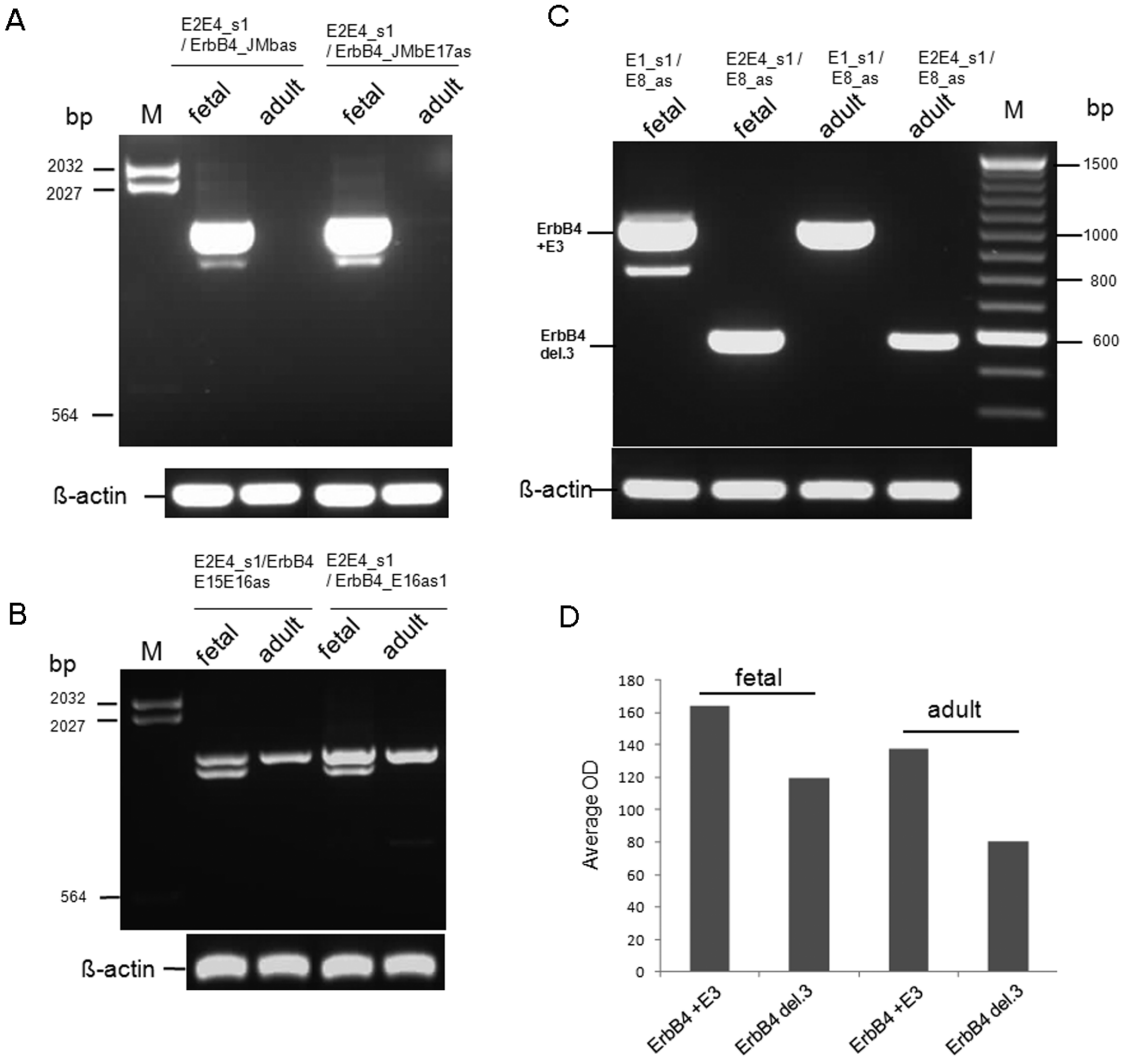
Expression of ErbB4 del.3/JM-b (A) and ErbB4 del.3 JM-a (B) splice isoform mRNAs in the adult and fetal human brain. For verification purposes, dual sets of primers were used for amplification of JM-a and JM-b (as labeled). Predicted amplicon sizes: ErbB4 del.3/JM-b (E2E4_s1 plus ErbB4_JMbas: 1489 bp, upper band and E2E4_s1 plus ErbB4_JMbE17as: 1518 bp, upper band). ErbB4 del.3 JM-a (E2E4_s1 plus ErbB4_E15E16as: 1478 bp, upper band and E2E4_s1 plus ErbB4_E16as1: 1484 bp, upper band). Of note, additional smaller bands were detected in the fetal brain for both del.3/JM-b and del.3/JM-a amplifications. (C) Semi-quantitative RT-PCR analysis of ErbB4 del.3 and ErbB4 +E3 mRNA abundance in adult and fetal human brain. Amplicon sizes ErbB4 +E3 (E1_s1 plus E8_as: 1011bp). ErbB4 del.3 (E2E4_s1 plus E8_as: 578bp). (D) Mean optical density (OD) values for ErbB4 del.3 and ErbB4 +E3 bands in adult and fetal brain. OD values for β-actin were equal across all samples.

RT-PCR analysis of ErbB4 del.3 mRNA expression in the adult and fetal human brain reveals that exon 3 skipping transcripts are readily detectable (Figure 4A and 4B). Consistent with the cloning data, ErbB4 del.3/JMb/CYT-2 isoforms are exclusively expressed in fetal brain and absent in the adult brain (Figure 4A). In contrast, we identified that ErbB4 del.3/JM-a/CYT-2 mRNAs are expressed in both adult and fetal brain (Figure 4B). The fact that we did not identify del.3/JM-a/CYT-2 in the PCR screen of the fetal brain library is likely due to the predominance of the del.3/JM-b transcript. These results combined with quantitative PCR (qPCR) analysis of overall JM-a and JM-b mRNA abundance in adult brain vs. fetal brain (data not shown), demonstrate that ErbB4 JM-b isoforms are differentially expressed in the fetal and adult human brain (JM-b, fetal > adult) and that this is likely due in part to the differential contribution of ErbB4 del.3 variants to the JM-b isoform pool.

Semi-quantitative RT-PCR analysis of total ErbB4 del.3 mRNAs and total ErbB4 exon 3 inclusive mRNAs (ErbB4 +E3) reveals that ErbB4 del.3 transcripts are relatively abundant in the fetal and adult brain (Figure 4C and 4D). In human fetal brain, the ratio of ErbB4 del.3 mRNAs to ErbB4 +E3 mRNAs is approximately 0.71∶1 and in adult brain 0.58∶1, respectively (Figure 4D). Consistent with the data described in Figure 4A and 4B, ErbB4 del.3 isoforms were more abundant in the fetal, compared to the adult human brain (Figure 4D; ratio 1.2∶1).

### Open reading frame (ORF) analysis of del.3/JM-b/CYT-2 transcripts

Open reading frame (ORF) analysis predicts that omission of the 187-nt exon 3 introduces an in-frame premature termination codon, UAA in exon 4. Introduction of a premature termination codon in the exon 4 is predicted to create a short ORF, encoding a C-terminal truncated peptide of 80 amino acids (Figure S5, see ORF1). An alternative AUG triplet exists downstream of exon 4 in exon 7 which could potentially be used to create a long ORF, giving rise to a protein of 1033 amino acids (JM-b; Figure S5, see ORF2) or 1043 amino acids (JM-a). N-terminal truncated ErbB4 proteins would have an identical C-terminal amino acid sequence to the corresponding wild-type ErbB4 JM-a or JM-b/CYT-2 protein.

## Discussion

In the present study, we have demonstrated that exon 3 of the human ErbB4 gene is a novel alternative exon, and that ErbB4 exon 3-skipping splice variants are abundant in human fetal and adult brain. Interestingly, ErbB4 del.3 transcripts are exclusively expressed as CYT-2 isoforms. Given the absence of identification of an ErbB4 del.3 CYT-1 transcript, we conclude that a mechanism of association of schizophrenia-risk polymorphisms (rs707284, rs839523 and rs7598440) to CYT-1 expression traits in the brain in schizophrenia [7] is unlikely related to exon 3 skipping.

In the adult brain, del.3/CYT-2 transcripts exist exclusively as JM-a variants and in the fetal brain as predominantly JM-b, with lower levels of JM-a. These results indicate that JM-a and JM-b isoforms are differentially expressed and regulated during human neurodevelopment. At present, the mechanism and function of exon 3 skipping is unknown. Inclusion of an alternative exon can be influenced by interaction between *trans*-acting splicing regulatory factors and *cis*-acting regulatory elements in exons or introns, such as exonic or intronic splicing enhancer/silencers and histone methylation [13]. Additional studies are underway to address these questions.

We observed in the human fetal brain that ErbB4 del.3 transcripts exist predominantly as JM-b/CYT-2 isoforms. A common feature of the above transcripts is that exon 16 is excluded, but a 45-nt sequence is included between exons 15 and 17. It has been previously shown that this sequence encodes amino acids specific for the ErbB4 JM-b isoform [9], however, this sequence was originally annotated to exon 15 [10]. Sequencing and mapping analysis to current builds of the human genome sequence reveal that this 45-nt sequence is located upstream of exon 16, but downstream of exon 15 of the ErbB4 gene. This 45-nt sequence comes from neither the exon 15 nor exon 16 and is located 121-nt upstream of exon 16, flanked by consensus splice sites. We propose to name the JM-b specific sequence, exon E15b, to distinguish it from exon 15.

Original cloning of human ErbB4 cDNA characterized the JM-a isoform as the inclusion of 23 amino acids (NGPTSHDCIYYPWTGHSTLPQHA) and the JM-b isoform as the inclusion of 13 alternative amino acids (IGSSIEDCIGLMD; [9], [10]). Our findings at the DNA sequence level for these variants are consistent to those reported previously [9], [10]. However, mapping to the current version of the human genome reveals that ErbB4 exon 16 actually encodes 24 amino acids (CNGPTSHDCIYYPWTGHSTLPQHA), and exon E15b encodes 14 amino acids (CIGSSIEDCIGLMD). Although the first amino acid from both the JM-a and JM-b isoforms is the same (cysteine), these are actually encoded by unique nucleotide sequences derived from different exons. Incomplete reference sequence information and limited details of exon-intron boundaries of the human ErbB4 gene at the time of the original cloning [9], likely contributed to the cysteine being attributed to the same upstream exon.

We clarify, therefore, that the ErbB4 gene consists of 29 exons; that exon 15 is constitutive, that exons 3, 15b, 16, and 26 are alternative and that 15b and 16 are mutually exclusive.

The potential functional significance of ErbB4 del.3 transcripts remains to be determined. ORF analysis predicts that omission of the 187-nt exon 3 results in the introduction of a premature termination codon in exon 4 creating a C-terminal truncated protein of 80 amino acids. An alternative translation initiator is present in exon 7 and predicted to generate a protein of 1033 amino acids. Previous data suggests that a prerequisite for downstream reinitiation of translation is the presence of a short upstream ORF [14]. Upstream ORFs longer than 165-nt completely abolish reinitiation of translation from a downstream ORF [14]. Because the first ORF in the ErbB4 del.3 transcript is 240-nt in length, it is unlikely that the second AUG in exon 7 would be used as an initiator for reinitiation of translation. Alternatively, the ‘leaky scanning mechanism’ is another way by which two proteins can be produced from a single mRNA transcript [15]. The leaky scanning model dictates that if the first AUG is in a less favorable sequence context, then a fraction of 40S ribosomal subunits will bypass the first AUG and initiate translation at a downstream AUG. Because the AUG in the ErbB4 exon 1 is in a good sequence context for translation (CAAAAA**AUG**A), due to an ‘A’ at the −3 position and +4 positions, respectively, it is predicted that almost all ribosomes would initiate translation at the first AUG in exon 1 Experimental verification of this process is necessary.

Taken together, our observations suggest that ErbB4 del.3 transcripts are likely to generate a C-terminal truncated protein of differential function to wild-type ErbB4. Alternatively, the premature termination codon may target these transcripts for nonsense mediated mRNA decay (NMD; [16]). However, the abundance of full length ErbB4 del.3 mRNA transcripts in human brain argues against such degradation. Future studies are required to determine the contribution of ErbB4 exon 3 skipping to human neurodevelopment and genetic diseases, but our findings suggest that ErbB4 del.3 transcripts exist as attractive potential modulators of ErbB4 signaling. Our findings overall have implications for studying and understanding the role of ErbB4 in the pathogenesis of human complex disease including neurodevelopmental disorders and cancer [6]–[8], [17].

## Materials and Methods

### Molecular Cloning and Sequencing of ErbB4 cDNA clones Derived from Adult and Fetal Human Brain Libraries

#### RT-PCR Amplification

To determine whether exon 3 of the ErbB4 gene is skipped in human brain, we used human fetal or adult whole brain Marathon-Ready cDNA library (Clontech, Mountain View, CA), respectively, as a template for PCR amplifications. Double-stranded cDNA clones were ligated at both ends to an adaptor that contains adaptor primer 1 (AP1) as well as adaptor primer 2 (AP2) sequences. To determine the expression of a del.3 transcript, we designed ErbB4 gene-specific reverse primers in exons 4 and 5, respectively. A nested PCR strategy was employed to screen for ErbB4 del.3 variants. Briefly, an outer primer pair [AP1 (5′-CCATCCTAATACGACTCACTATAGGGC-3′) and ErbB4E5_as(5′-GTCCCCAGCAACGGCCAGTACA- 3′)] was used in the first-round PCR, and subsequently an inner primer pair [AP2 (5′-ACTCACTATAGGGCTCGAGCGGC-3′) and ErbB4E4_as(5′-GAACTACCATTTGTTGACACAAGAGT-3′)] was used in the second-round PCR. First- and second-round PCRs were performed in a 50-µl final reaction volume using the Platimun High Fidelity Taq DNA polymerase (Invitrogen, Carlsbad, CA). PCR amplifications were as follows: denaturation for 1 min at 94°C, annealing for 2 min at 60°C, and extension for 2 min at 68°C, with a total of 35 cycles. For the last cycle, the PCR products were further extended for 10 min at 68°C for completion.

To determine whether ErbB4 exon 3 skipping (del.3) occurs on the CYT-1 or CYT-2 isoform background of full length-ErbB4, we designed a specific forward primer, E2E4_s1(5′-GTTCCTGCGGAAATCCTAAATGGTG-3′) that spans the junction of exons 2 and 4 of the ErbB4 gene, and two reverse primers located in the penultimate exon 27. Using a human fetal brain Marathon-Ready cDNA library as a template, we performed first-round PCR using an outer primer pair AP1 and reverse primer E27_as1(5′-GTATTCTTGTTTGGGTTTGTCTCGCATAGGA-3′). The second-round PCR was conducted by using an inner primer pair E2E4_s1 and E27_as2 (5′- GCAGCAAAACCTCCATCTCGGTATA-3′). An adult human whole brain Marathon-Ready cDNA library was used as a template for amplification of ErbB4 del.3 variants in the adult brain. The first-round PCR was performed by using an outer primer pair AP1 and E28_as1 (5′-GACCAACCCATGCAGAGAAATGAA- 3′). The second-round PCR was conducted by using an inner primer pair E2E4_s1 and E27_as2.

#### cDNA cloning and sequencing

Amplified PCR products were separated by electrophoresis on 1% agarose gel in 0.5x TBE, stained with ethidium bromide and visualized under ultraviolet light. DNA bands were dissected from the gel, and purified by the QIAquick Gel Extraction Kit (QIAGEN, Valencia, CA). Purified DNA fragments were cloned into either pCR@-XL-TOPO vector or pCR@2.1-TOPO vector using the TA-Cloning Kit (Invitrogen, Carlsbad, CA). Two microliters of the TOPO cloning reaction was used to transform a bacterial strain TOP10 and the resulting colonies were grown in cultures overnight at 37°C at 225 rpm in a shaker. The plasmid DNAs were isolated by using the QIAprep Spin Miniprep Kit (QIAGEN, Valencia, CA). The BigDye Terminator Kit (Applied Biosystems, Foster City, CA) was used to determine the sequence of an insert in each plasmid with either the M13F primer (5′-GTAAAACGACGGCCAG-3′) or the M13R primer (5′-CAGGAAACAGCTATGAC-3′). For ErbB4 del.3 transcripts longer than 2 kb, additional primers covering several constitutive exons of the ErbB4 gene were used for deeper sequencing of the inserts (Table 1).

**Table 1:** Primers used for sequencing of ErbB4 cDNA clones.

Names of primers	Primer sequences (5 --- 3)
E4_s	GGAGTCTATGTAGACCAGAACAAA
E8_s	GTAGAAGAAAATGGGATTAAAATGTGT
E12_s	GAATAGTAATCCGGGACAACAGAAAA
E17_s	GTTAGAAGGAAGAGCATCAAAAAGAAA
E21_s	GGAAGGAGATGAAAAAGAGTACAAT
E5_as	GTCCCCAGCAACGGCCAGTACA
E9_as	GACTAGAAAGATCAAATTCCCATTGAT
E13_as	GAGGTTACAAGACTCTATGCAGAT
E18_as	GAGCCAAGGACTTTTACCCTCTT
E22_as	AACGTCACTCTGATGGGTGAATTT

#### Analysis of ErbB4 del.3 JM-a/JM-b mRNA isoform expression and ErbB4 del.3 mRNA abundance in adult and fetal brain

To determine whether ErbB4 del.3 transcripts with JM-a or JM-b coding regions are differentially expressed between the human fetal and adult brain, we used Marathon-Ready fetal or adult whole brain cDNA as a template, respectively, and nested PCR. The first round of PCR was conducted by using the primer pair AP1 and E28-as1 [5′ -GACCAACCCATGCAGAGAAATGAA- 3′]. Five microliters of 1∶100 diluted first-round PCR products was used in the second-round PCRs in a total volume of 50 µl reaction. To amplify del.3/JM-b specific splice isoforms, two primer pairs were used, respectively, in the second-round PCRs (E2E4_s1 plus ErbB4_JMbas, and E2E4_s1 plus ErbB4_JMbE17as; see Table S1). To amplify del.3/JM-a specific splice isoforms, two primer pairs were used, respectively (E2E4_s1 plus ErbB4_E15E16as and E2E4_s1 plus ErbB4_E16as1; see Table S1). Loading of gels comprised 6 ul per sample for del.3/JM-b/CYT-2 mRNA (Figure 4A) and 20 ul per sample for del.3/JM-a/CYT-2 mRNA (Figure 4B).

To determine the relative abundance of ErbB4 del.3 mRNAs we performed semi-quantitative RT-PCR using primer pairs in exon 1 (E1_s1) and exon 8 (E8_as) to amplify ErbB4 + E3 mRNAs and primer pairs spanning exon 2-exon 4 (E2-E4_s1) and exon 8 (E8_as, see Table S1) to amplify all ErbB4 del.3 mRNAs. PCR conditions were as described above. All PCR amplifications were carried out under identical conditions, on the same PCR plate and during the same reaction (β-actin was used as an internal control). Optical density (OD) values were determined using appropriate software (NIH Image J version 1.43, U.S. National Institutes of Health). β-actin was measured as an internal control in all experiments. The primers used for amplification of β-actin were bactin_E1E2s [5′-CGCCGCCAGCTCACCATGGAT-3′] and bactinE3E4as [5′-TCTCAAACATGATCTGGGTCATCTT-3′].

## Supporting Information

Figure S1 Schematic illustration of an ErbB4 del.3 transcript of the human ErbB4 gene. A. The 5′ region of the exon-intron structure of human ErbB4 gene is shown. Open boxes represent exons. The horizontal lines represent the introns. A three SNP haplotype associated with increased risk for schizophrenia is shown with rs numbers, respectively. B. An ErbB4 del.3 transcript cloned from human fetal brain. Open boxes represent exons.
(1.43 MB TIF)

Figure S2 Nested PCR amplification of ErbB4 Del.3 transcripts from fetal (A) and adult (B) human brain cDNA libraries. A. A 2.73 kb amplicon derived from fetal brain using a forward primer, (E2E4_s1), spanning the junction of exons 2 and 4 combined with reverse primers in exon 27. B. 2.76 kb amplicon derived from adult brain using the forward primer, E2E4_s1 and reverse primers in exon 27. S (sample), M (lambda DNA/HindIII Marker).
(0.85 MB TIF)

Figure S3 Raw sequence chromatograms showing the JM-b and JM-a exon boundaries.
(7.36 MB TIF)

Figure S4 ErbB4 del.3 is expressed as a JM-a/CYT-2 isoform in the human adult brain. Open boxes represent exons. The horizontal lines with the first “//” sign represent exons E6 to E14, whereas the horizontal lines with the second “//” sign represent exons E18 to E23. The PCR primers are indicated as forward or reverse bent arrows, respectively. The three skipped exons (exons 3, 15b, and 26) are indicated. The 24 amino acid encoded by exon 16, specific for the JM-a isoform, is depicted.
(4.78 MB TIF)

Figure S5 Predicted potential open reading frames (ORFs) for translation from the ErbB4 exon 3-skipping JM-b/CYT-2 isoform. Nucleotide positions (−3 and +4) potentially affecting efficiency of translation initiation around an AUG codon are indicated. The position of an “A” in the AUG is assigned as +1. Open boxes represent exons. The horizontal lines with the first “//” sign represent the ErbB4 exons from E8 to E14, whereas the horizontal lines with the second “//” sign represent exons E18 to E23.The three skipped exons (exons 3, 16, and 26), as well as JM-b isoform specific exon 15b are indicated. Nucleotide sequences around the AUG in exons 1 and 7 are shown. Potential ORFs (ORF1 and ORF2) are indicated with two-head arrows, as well as the length of the potentially encoded amino acids.
(5.39 MB TIF)

Table S1 Primers for RT-PCR amplifications of ErbB4 del.3 JM-a/JM-b, ErbB4 del.3 and ErbB4 +exon3 mRNAs in human fetal and adult brains. Abbreviations: E, exon: s, sense and as, antisense.
(0.04 MB DOC)
