# Supplementary figures and images for: Molecular Cloning and Characterization of the Human ErbB4 Gene: Identification of Novel Splice Isoforms in the Developing and Adult Brain
Source: PLoS One. 2010 Sep 23;5(9):e12924. doi: 10.1371/journal.pone.0012924 (PMC2944867; doi:10.1371/journal.pone.0012924)

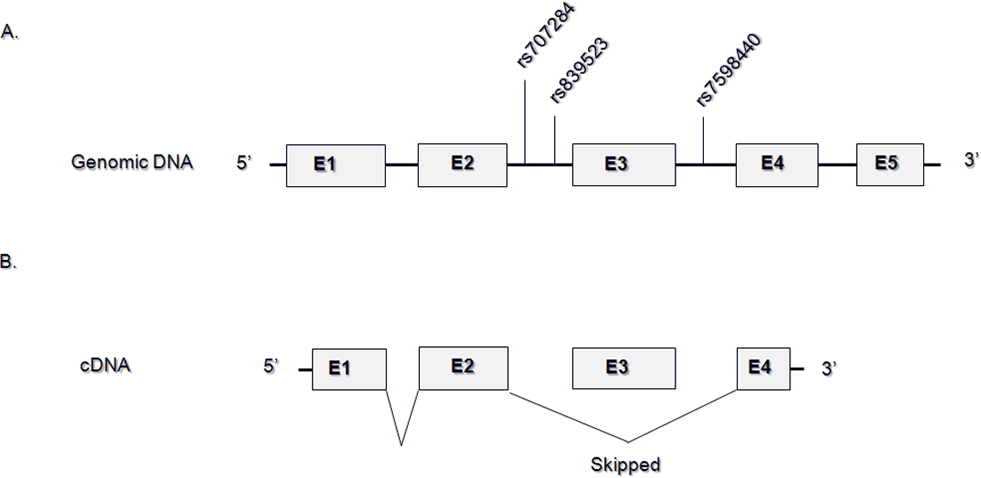

Supplement: Figure S1 — Schematic illustration of an ErbB4 del.3 transcript of the human ErbB4 gene. A. The 5′ region of the exon-intron structure of human ErbB4 gene is shown. Open boxes represent exons. The horizontal lines represent the introns. A three SNP haplotype associated with increased risk for schizophrenia is shown with rs numbers, respectively. B. An ErbB4 del.3 transcript cloned from human fetal brain. Open boxes represent exons. (1.43 MB TIF) [file pone.0012924.s001.tif]

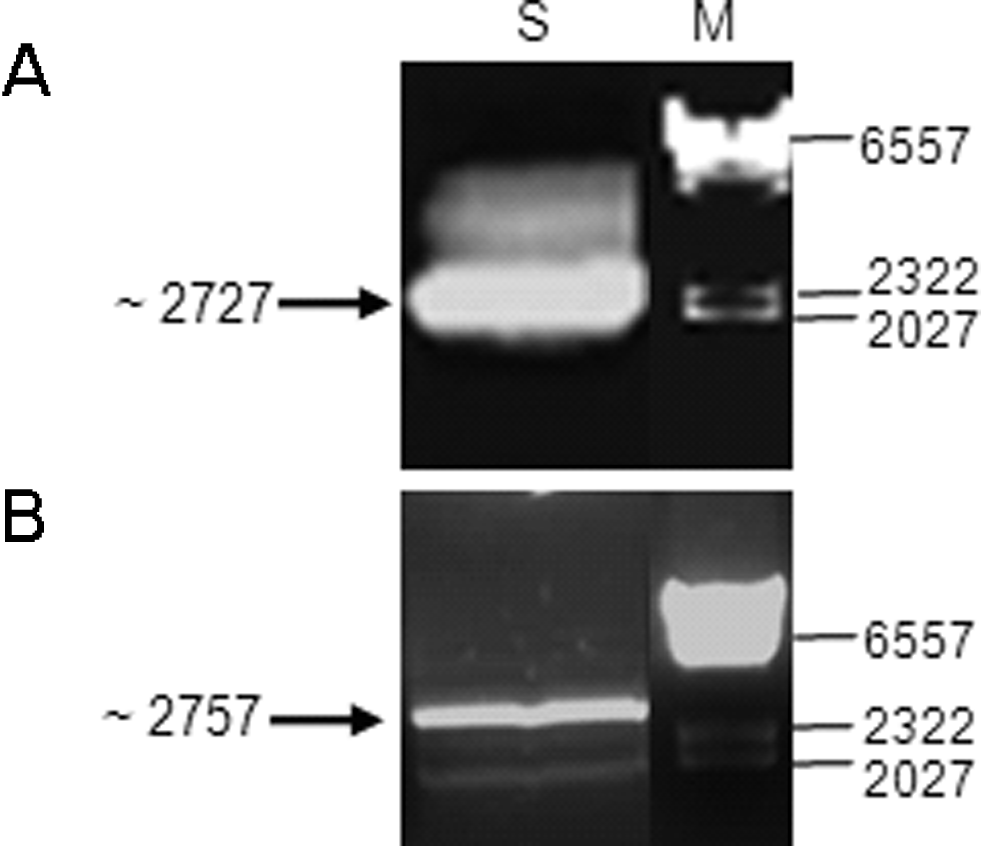

Supplement: Figure S2 — Nested PCR amplification of ErbB4 Del.3 transcripts from fetal (A) and adult (B) human brain cDNA libraries. A. A 2.73 kb amplicon derived from fetal brain using a forward primer, (E2E4_s1), spanning the junction of exons 2 and 4 combined with reverse primers in exon 27. B. 2.76 kb amplicon derived from adult brain using the forward primer, E2E4_s1 and reverse primers in exon 27. S (sample), M (lambda DNA/HindIII Marker). (0.85 MB TIF) [file pone.0012924.s002.tif]

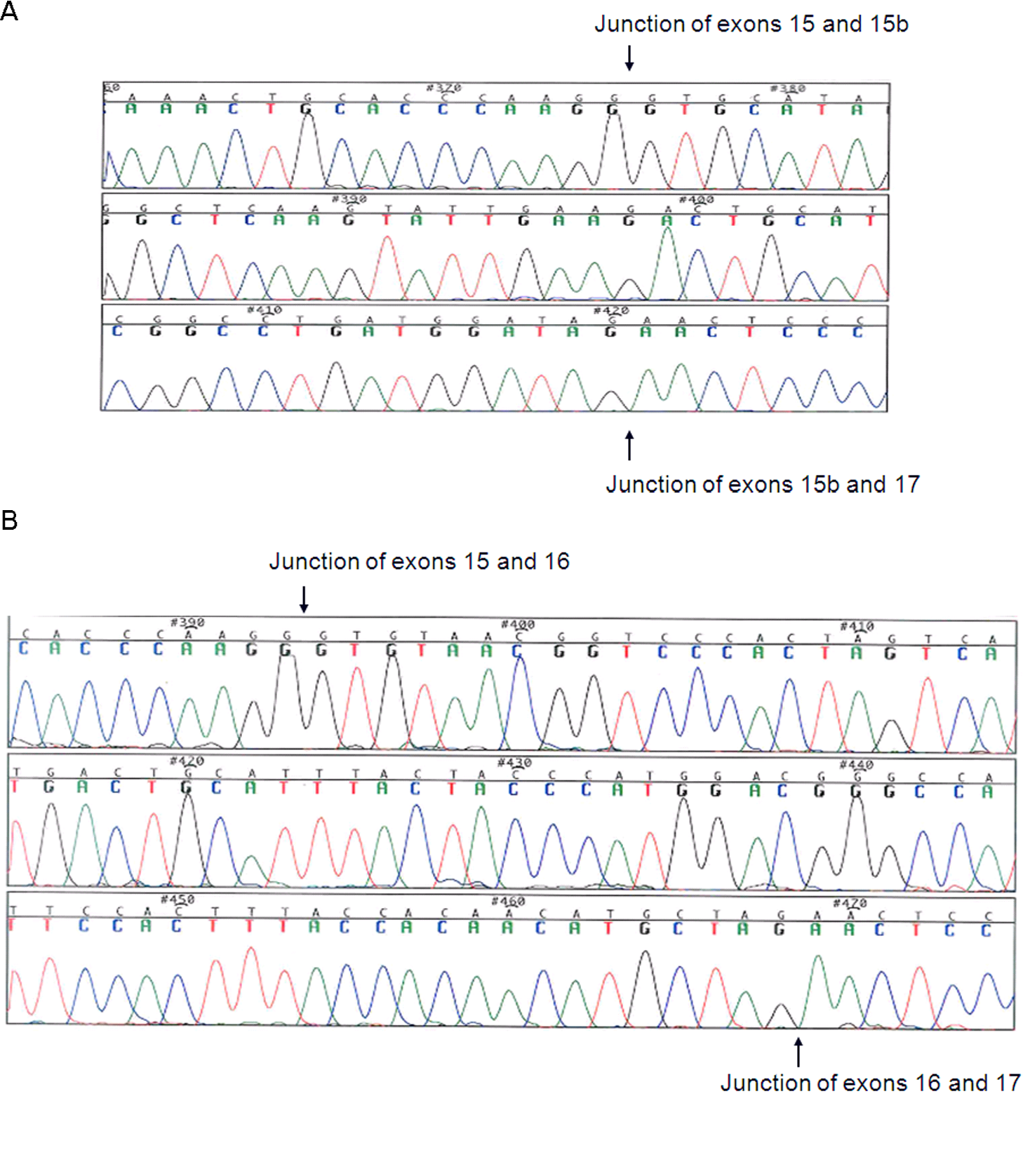

Supplement: Figure S3 — Raw sequence chromatograms showing the JM-b and JM-a exon boundaries. (7.36 MB TIF) [file pone.0012924.s003.tif]

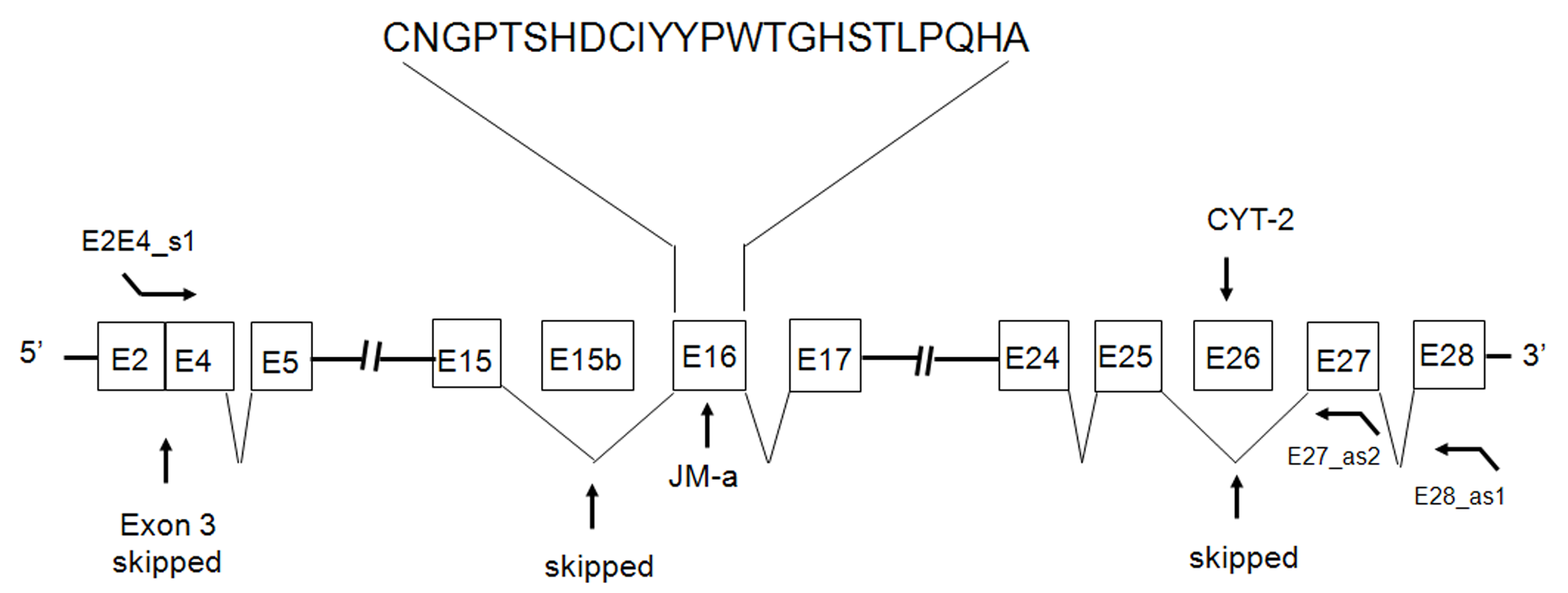

Supplement: Figure S4 — ErbB4 del.3 is expressed as a JM-a/CYT-2 isoform in the human adult brain. Open boxes represent exons. The horizontal lines with the first “//” sign represent exons E6 to E14, whereas the horizontal lines with the second “//” sign represent exons E18 to E23. The PCR primers are indicated as forward or reverse bent arrows, respectively. The three skipped exons (exons 3, 15b, and 26) are indicated. The 24 amino acid encoded by exon 16, specific for the JM-a isoform, is depicted. (4.78 MB TIF) [file pone.0012924.s004.tif]

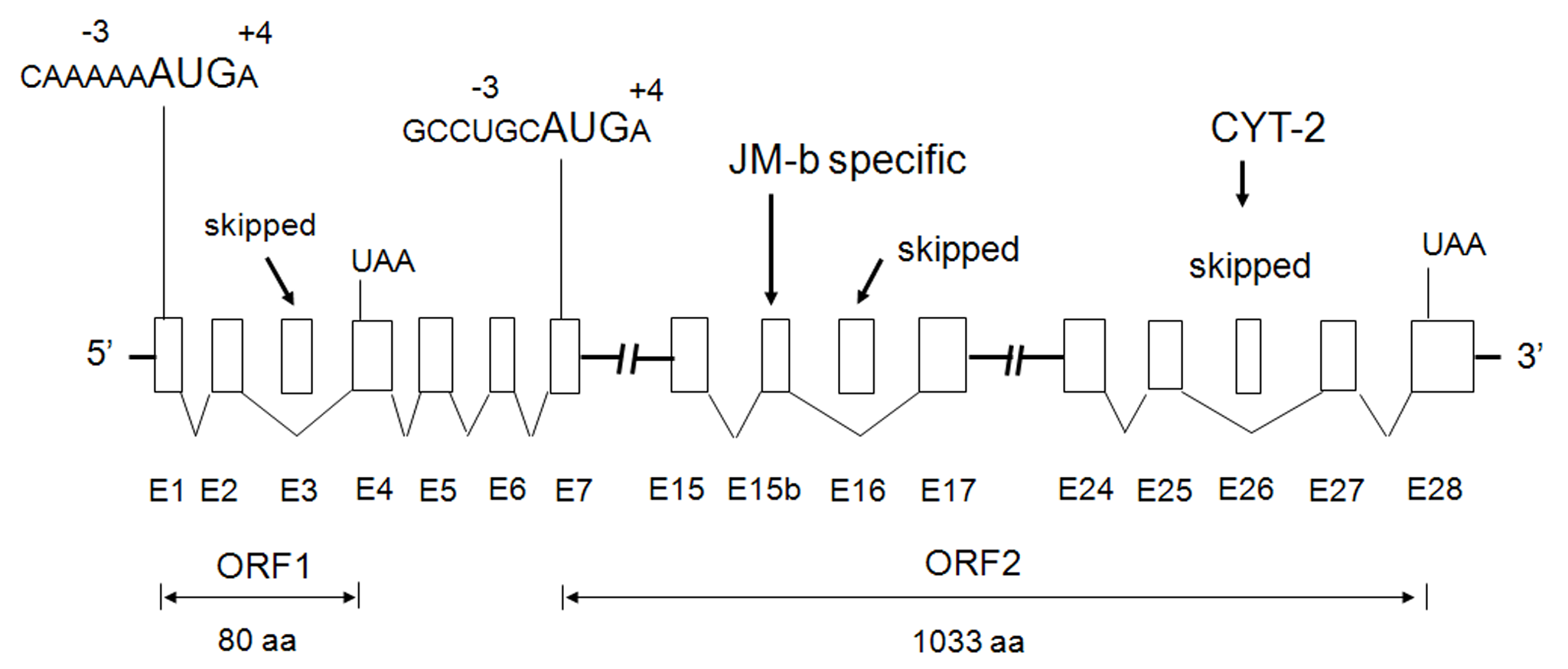

Supplement: Figure S5 — Predicted potential open reading frames (ORFs) for translation from the ErbB4 exon 3-skipping JM-b/CYT-2 isoform. Nucleotide positions (−3 and +4) potentially affecting efficiency of translation initiation around an AUG codon are indicated. The position of an “A” in the AUG is assigned as +1. Open boxes represent exons. The horizontal lines with the first “//” sign represent the ErbB4 exons from E8 to E14, whereas the horizontal lines with the second “//” sign represent exons E18 to E23.The three skipped exons (exons 3, 16, and 26), as well as JM-b isoform specific exon 15b are indicated. Nucleotide sequences around the AUG in exons 1 and 7 are shown. Potential ORFs (ORF1 and ORF2) are indicated with two-head arrows, as well as the length of the potentially encoded amino acids. (5.39 MB TIF) [file pone.0012924.s005.tif]
