# Supplementary material for: Molecular Cloning and Characterization of the Human ErbB4 Gene: Identification of Novel Splice Isoforms in the Developing and Adult Brain
Source: PLoS One. 2010 Sep 23;5(9):e12924. doi: 10.1371/journal.pone.0012924 (PMC2944867; doi:10.1371/journal.pone.0012924)
Supplement: Table S1 — Primers for RT-PCR amplifications of ErbB4 del.3 JM-a/JM-b, ErbB4 del.3 and ErbB4 +exon3 mRNAs in human fetal and adult brains. Abbreviations: E, exon: s, sense and as, antisense. (0.04 MB DOC) [file pone.0012924.s006.doc]

Names of primers Primer sequences (5’ --- 3’)

E2E4_s1 GTTCCTGCGGAAATCCTAAATGGTG

ErbB4_JMbas GATGCAGTCTTCAATACTTGAGCCTA

ErbB4_JMbE17as GCAATCAGGGGAGTTCTATCCATCA

ErbB4_E15E16_as ACTAGTGGGACCGTTACACCCTTG

ErbB4_E16as1 GTCATGACTAGTGGGACCGTTACA

ErbB4_E16as2 GAATGGCCCGTCCATGGGTAGTA

ErbB4_E16E17as GCAATCAGGGGAGTTCTAGCATGTT

E1_s1 GGATCTGAGACTTCCAAAAAATGAA

E8_as GGCAAATGTCAGTGCAAGGTTTACA
